# L-Glutamine Supplementation Improves the Benefits of Combined-Exercise Training on Oral Redox Balance and Inflammatory Status in Elderly Individuals

**DOI:** 10.1155/2020/2852181

**Published:** 2020-01-22

**Authors:** Ewin B. Almeida, Juliana M. B. Santos, Vitória Paixão, Jonatas B. Amaral, Roberta Foster, Adriane Sperandio, Tamaris Roseira, Marcelo Rossi, Telma G. Cordeiro, Fernanda R. Monteiro, Gislene R. Amirato, Carlos A. F. Santos, Rodolfo P. Vieira, Mauro Vaisberg, Marcelo P. Barros, André L. L. Bachi

**Affiliations:** ^1^Department of Otorhinolaryngology, ENT Lab, Federal University of São Paulo (UNIFESP), Rua dos Otonis, 700, Piso superior/Second floor, Sao Paulo SP 04025-002, Brazil; ^2^Method Faculty of Sao Paulo (FAMESP), Av. Jabaquara, 1314, Sao Paulo SP 04046-200, Brazil; ^3^Brazilian Institute of Teaching and Research in Pulmonary and Exercise Immunology (IBEPIPE), Rua Pedro Ernesto 240, São José dos Campos, SP 12245-520, Brazil; ^4^Post-graduation Program in Bioengineering and Biomedical Engineering, Universidade Brasil, Rua Carolina Fonseca 584, São Paulo, SP 08230-030, Brazil; ^5^Post-graduation Program in Science of Human and Rehabilitation, Federal University of São Paulo (UNIFESP), Av. Ana Costa 95, Santos, SP 11060-001, Brazil; ^6^School of Medicine, Anhembi Morumbi University, Av. Dep. Benedito Matarazzo 6070, São José dos Campos, SP 12230-002, Brazil; ^7^Interdisciplinary Postgraduate Program in Health Sciences, Institute of Physical Activity Sciences and Sports (ICAFE), Cruzeiro do Sul University, Rua Galvão Bueno 868, São Paulo, SP 01506-000, Brazil

## Abstract

Although regular combined aerobic-resistance exercises can ameliorate the inflammatory status and redox balance in elderly population, it is unclear whether protein or specific amino acid supplementation could improve such benefits. Therefore, we aimed to evaluate the inflammatory status and redox indexes through of the saliva of 34 elderly subject nonpractitioners (NP group, 73.3 ± 6.6 years) and 49 elderly subject practitioners of a combined-exercise training in moderate intensity (CET group, 71.9 ± 5.8 years) before (pre) and after (post) 30 days of supplementation with L-glutamine (Gln) or placebo (PL). Our results showed that, both in pre- and postsupplementation, the salivary levels of nitric oxide (NO^·^) and TNF-*α* were lower, whereas the levels of uric acid and IL-10 (as well as IL-10/TNF-*α* ratio) were higher in the CET groups than in the NP groups. In postsupplementation, both groups supplemented with Gln (NP-Gln and CET-Gln) showed higher salivary uric acid levels compared to baseline. In addition, lower NO^·^ levels were found in the CET-Gln group postsupplementation than presupplementation values. Whereas the CET-Gln group showed lower GSH levels postsupplementation, NP-Gln subjects showed lower GSSG levels at the same time point, both compared to baseline. Interestingly, salivary peroxidase activity was lower only in NP groups (NP-PL and NP-Gln) postsupplementation than baseline values. A positive significant correlation between salivary peroxidase activity and GSH levels, and also between salivary peroxidase activity and uric acid levels were observed in the CET-Gln group both pre- and postsupplementation. No differences were found in albumin, total antioxidant activity (TEAC), and reducing power analysis between groups, pre- or postsupplementation. In conclusion, the elderly subjects from the CET group showed a better inflammatory response and redox balance and, for the first time, it was shown that daily supplementation with Gln for 30 days can improve these benefits with putative association with a healthy aging.

## 1. Introduction

The World Health Organization (WHO) has estimated that the proportion of elderly population around the world will grow from 12% to 22% between 2015 and 2050, showing a faster increase of this population compared to any other age range [1]. This accelerated pace of elderly population grow can be attributed to an expressive improvement of primary health care and financial investments in programs of healthy aging promotion. However, the dark side of this scenario is the significant elevation in the incidence of diseases and comorbidities closely associated with the aging process [2].

It is widely accepted that aging is a natural physiological process characterized by declines in the function of several body systems, leading to a severe alteration in key homeostatic systems, such as the redox balance and inflammatory control [3]. Regarding the relationship between aging and inflammatory processes, the term “inflammaging” has been currently used to describe a low-grade chronic systemic inflammation associated with aging [4, 5]. The occurrence of inflammaging, with higher circulating levels of tumor necrosis factor-alpha (TNF-*α*) and interleukin- (IL-) 6, two proinflammatory cytokines, is associated with the development of many diseases observed in the elderly population [6].

According to the literature, chronic elevations of proinflammatory cytokines might be linked with an increase of oxidative stress, which also impacts negatively on healthy aging [7]. Higher oxidative conditions imposed by elevated production of reactive oxygen and nitrogen species (ROS and RNS, respectively) and depletion of antioxidant defenses (as regularly observed during the aging process), in association with enhanced proinflammatory responses, can severely lead to redox imbalances in many body fluids, cells, tissues, and organs of the elderly population, triggering other redox-based comorbidities [8].

In an attempt to minimize the imbalance of both inflammation and oxidative stress associated with aging, some nonpharmacological interventions, such as nutritional care and regular practice of physical exercises, are well accepted and largely recommended. Indeed, it has been demonstrated that the moderate exercise training has beneficial effects on the systemic inflammatory response in elderly individuals, not only by reducing proinflammatory cytokines, as TNF-*α*, IL-1*β*, and IL-6 [9] but also by increasing the circulating levels of IL-10, a classic anti-inflammatory cytokine [10, 11]. Noteworthy, the American College of Sports Medicine (ACSM) states that training programs must contain combined exercises (endurance and resistance) for maximum benefits in human health, by optimizing cardiovascular, respiratory, immune, and muscle functions. Moreover, practitioners of combined-exercise training also improve the acquisition, maintenance, and recovery of their physical capacities, which were lost during aging [12, 13].

As aforementioned, nutritional care can also be used as a good strategy to minimize the deleterious effects of aging, not only in individuals who cannot engage into regular exercise training programs but also to promote the effects of moderate exercises in elderly subjects [14]. For instance, amino acid supplementation can act in maintenance or even increase of muscle mass, with a adjutant role in the regulation of inflammatory and antioxidant responses in the elderly population [15–17]. In this context, glutamine (Gln) has an essential role in the synthesis of glutathione (GSH), a renown endogenous antioxidant, and in the systemic nitrogen metabolism and transport between organs [16]. Furthermore, Gln may suppress the production of proinflammatory cytokines, such as TNF-*α*, improving the control of inflammation [18]. Even though Gln is the most abundant and versatile amino acid in the body, it is classified as a conditionally essential amino acid because under some stress situations (or during aging), the human body reduces the ability to synthesize sufficient quantities of this amino acid, which can compromise many body and cellular functions [19].

In order to evaluate the redox balance and inflammatory response, the use of the saliva is a powerful and noninvasive tool linked not only with oral hygiene and buccal disorders but also with the general health state of humans [20, 21] . Furthermore, the evaluation of redox/inflammatory biomarkers in saliva was proposed as an accurate diagnosis for the development and progress of many oxidative stress-mediated diseases, such as diabetes [22], rheumatoid arthritis [23], chronic renal failure [24], obesity [25], and many neurodegenerative disorders [26]. Not surprisingly, the impairment of such important salivary enzymes could drastically hamper energy metabolism and both oral and gastrointestinal microbiota functions, increasing the risk of infectious diseases [27].

Based on that, we aim here to investigate, through the saliva, the effect of both combined-exercise training and Gln supplementation on inflammatory response and redox imbalance in elderly subjects.

## 2. Materials and Methods

### 2.1. Subjects and Study Design

**Figure 1:**
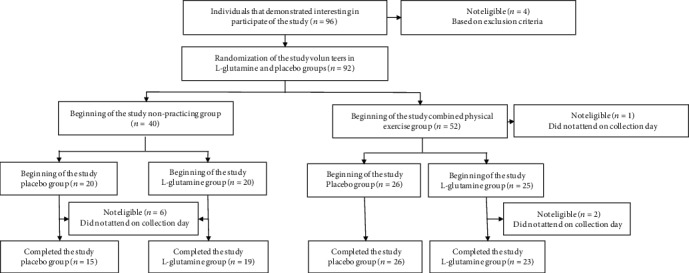
Flow diagram of the study.

As demonstrated in the flow diagram (Figure 1), eighty-three elderly subjects (aged 72.4 ± 6.1), both men (*n* = 18) and women (*n* = 65), were enrolled in the study. Volunteers were recruited from the primary health care program belonging to the Geriatrics and Gerontology Discipline of the Federal University of São Paulo (UNIFESP). Based on a nutritional record evaluation, subjects were not taking any antioxidant/multivitamin supplements at the time of the study and their diets did not contain more than 4,000 cal/day or a protein intake >1.75 g/kg body mass (exclusion criterion 1), which could mask the effects of glutamine supplementation. The subjects attested to no use of anti-inflammatory drugs in the last two months (exclusion criterion 2). All the subjects were informed of the risks and benefits of the study prior to any data collection and gave written informed consent for their participation. The participants were oriented to report any noticed abnormality in their buccal health during the study. Volunteers were separated into two groups: non-practitioners (NP, *n* = 34; men = 8 and women = 26) and combined-exercise training subjects (CET, *n* = 49; men = 10 and women = 39). All volunteers signed the informed consent form previously approved by the Ethics Committee of the Federal University of São Paulo (approval number 3.623.247) and by the National Research Ethics Committee (number CAEE:218170619.3.0000.5505). The study was in agreement with the Ethical Standards of exercise practice, as well as all experiments were performed in accordance with the Declaration of Helsinki.

Clinical and physical examinations were carried out at the Geriatrics and Gerontology Discipline of the Federal University of São Paulo (UNIFESP) and none of the volunteers recruited presented type I diabetes mellitus, neoplasia, chronic infections, neurological, renal, and/or liver diseases, thrombosis, and cardiovascular or other diseases which precluded the performance of physical activity. Anthropometric characteristics (measurement of weight, height, and body mass index (BMI)) and also body composition by bioimpedance (BIOSCAN 920-2-S Maltron International Limited, UK) were assessed. Daily nutritional consumption of proteins and antioxidants was evaluated by the food frequency questionnaire. Physical activity level was assessed through the International Physical Activity Questionnaire (IPAQ), validated for the Brazilian population [28, 29].

### 2.2. L-Glutamine and Placebo Supplementation

**Figure 2:**
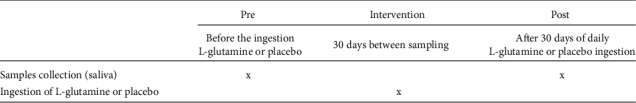
Experimental design.

Volunteers of NP and CET groups were randomly separated into 2 subgroups: NP-placebo (NP-PL, *n* = 15); NP-L-glutamine (NP-Gln, *n* = 19); CET-placebo (CET-PL, *n* = 26); and CET- L-glutamine (CET-Gln, *n* = 23). The groups supplemented with L-glutamine ingested 0.3 g/kg/day of L-glutamine (Tongliao Meihua Biological Sci Tech Co. Ltd., China) added to 10 g/day of maltodextrin (PR Netto Indústria e Comércio de Alimentos Ltda., SP, Brazil). The groups supplemented with placebo ingested 10 g/day of maltodextrin. All the volunteers were oriented to dilute 1 sachet of the supplement (L-glutamine or placebo) in 250 mL of water and ingest the solution immediately, for 30 consecutive days (Figure 2). In addition, participants were oriented to immediately interrupt this ingestion if any gastrointestinal disturbance was perceived. The dose of L-glutamine used here was based on the study of Legault et al. [30].

### 2.3. Combined-Exercise Training (CET)

Volunteers from the combined-exercise training (CET) group maintained a usual exercise routine consisting of the following exercise program: 3 workout days with 1 hour of training each day, performed for at least 12 months. Throughout the study, all participants were supervised by the same physical education professional to ensure the continuity of the CET.

The combined-exercise training program consisted of moderate aerobic exercises (ranging between 60 and 70% of the maximal heart rate reserve (MHHR), calculated by the equation (208 − (0.7 × age)), proposed by Tanaka et al. [31]) associated with localized resistance exercises (strength training), ranging from 50 to 60% of one-repetition maximum (1RM) test. Each session of aerobic training was performed within 30 minutes, both with exercises to improve postural stabilization and rhythm (with dance classes), as well as exercises performed on equipment, such as step platforms and trampolines. All physical exercises were performed with low impact and the training intensity was assessed by means of heart rate monitor (Polar brand, model FT1, Polar-Finland).

Resistance training involved 5 to 10 different exercises for the following muscle groups: lower and upper limbs, abdomen, buttocks, and those related to postural stabilization, including dorsal and lumbar spine muscles. The exercises were performed using dumbbells or body weight, in 2 sets of 10 to 20 repetitions, 30 minutes a day, soon after the end of aerobic training. Resistance training was applied 2 to 3 times a week and each training session involved different combinations of two muscle groups described above and was performed on 4 consecutive weeks. The intensity of resistance training was assessed using the Borg Scale of Perceived Exertion [32].

### 2.4. Collection of Samples

Saliva samples were obtained at two different occasions: before and after 30 days of daily supplementation. In relation to CET group, the volunteers were instructed to perform the last exercise session 24 hours before saliva collection.

Two milliliters (2 mL) of samples were collected directly, without prior stimulation or use of any collection material, in sterile 15 mL Falcon® tubes. The collected material was refrigerated at 4°C for 15 minutes and then centrifuged at 3000 rpm for 5 minutes. Then, 400 *μ*L of supernatant were added to 1.5 mL microtubes and kept frozen at -80°C, without any buffer or preservative added, for further analysis of cytokine concentration and antimicrobial peptides. Saliva samples containing blood were discarded.

### 2.5. Cytokine Determination

Cytokine concentrations were determined in the saliva samples by ELISA test. Biomarkers were: tumor necrosis factor alpha (TNF-*α*) (R&D System, Minneapolis, MN, USA) and interleukins-10 (IL-10) (Invitrogen by Thermo Fisher Scientific, Vienna, Austria) following the manufacturer's instructions. Concentration of cytokines was calculated using appropriate standard curves (following instructions from manufacturers). The linearity of ELISA methods of IL-10 and TNF-*α* was, respectively, within the 25–600 pg/mL and 6–1000 pg/mL ranges, which include the range of sample determinations. All correlation coefficients of standard curves were in the range of 0.95 to 0.99, whereas intra-assay coefficients of variance were 3–5%, and interassay coefficients of variance were 8–10%. Saliva cytokine concentration values were normalized by the total protein concentration determined by the Bradford method [33]. We also calculated IL-10/TNF-*α* ratios in order to estimate the progression of inflammatory processes in the biological samples [34].

### 2.6. Determination of Biochemical Indexes

Biochemical indexes were determined in samples previously stored. Albumin (Labtest Diagnostics S.A, Minas Gerais, MG, Brazil), uric acid (Labtest Diagnostics S.A, Minas Gerais, MG, Brazil), *α*-amylase (Biotécnica IND.COM. LTDA; Varginha, MG, Brazil), and NO· (kit EMSNO, Invitrogen™, Carlsbad, CA, US) were analyzed using colorimetric commercial kits following the manufacturer's instructions. Intra- and interassay coefficients of variance were 3-5% and 5-7.5%, respectively.

### 2.7. Determination of Trolox-Equivalent Antioxidant Capacity (TEAC)

TEAC was evaluated following the methodology described by Van den Berg et al. [35], using a radical solution of 2,2′-azinobis-(3-ethylbenzthiazoline-6-sulphonate) (ABTS^·-^) obtained by mixing 2.5 mM of 2,2′-azobis-(2-amidinopropane). HCl (ABAP) with 20 mM of ABTS stock solution diluted in 100 mM phosphate buffer (PBS, pH 7.4) containing 150 mM NaCl. The solution was heated for 12 min at 60°C, protected from light, and stored at room temperature. TEAC determination was performed as described by Leite et al. [36]. Intra- and interassay coefficients of variance were 3-5% and 7-12%, respectively.

### 2.8. Reduced/Oxidized Glutathione (GSH/GSSG)

Reduced (GSH) and oxidized (GSSG) glutathione content in the saliva were determined using colorimetric commercial kits (Bioassay System, Hayward, CA, USA) following the manufacturer's instructions. Intra- and interassay coefficients of variance were 3-5% and 8-10%, respectively.

### 2.9. Reducing Power of Saliva

The reducing power of the saliva was calculated based on GSH and GSSG contents and applying the 1 : 2 stoichiometry of thiol group oxidation, as follows [36]:

(1) $$\begin{array}{c} \text{Reducing power}=\left[\text{GSH}\right]/\left\{\left[\text{GSH}\right]+2\left[\text{GSSG}\right]\right\}. \end{array}$$

### 2.10. Statistical Analysis

Continuous and semicontinuous data were initially compared with the Gauss curve and the normality for each determined by the Kolmogorov-Smirnov test. The homogeneity of variance was verified by the Levene test.

Parametric variables were presented as mean and standard deviation, or standard error, ($\dot{\text{x}}\pm \text{SD}$ or $\dot{\text{x}}\pm \text{SE}$), and were statistically compared by 2-way ANOVA test for repeated measures with Student-Newman-Keus post hoc tests for intragroup comparison. Person's correlation coefficient test was applied for correlations between biochemical indexes. The *α* risk considered, for statistical differences, was ≤0.05 in this study (*p* ≤ 0.05).

## 3. Results

Table 1 shows the physical characteristics of the volunteers from NP and CET groups supplemented with placebo (NP-PL and CET-PL) or L-glutamine (NP-Gln and CET-Gln). No differences were observed in the age and height between the groups. However, the volunteers of NP group supplemented with Gln presented higher weight and, consecutively, BMI than the other groups. In relation to the bioimpedance analysis of total body fat, fat-free mass, skeletal muscle mass, and weekly physical activity levels by IPAQ, significant differences between the volunteers was observed. NP groups showed not only lower weekly physical activity levels but also higher weekly sit position time than the CET groups. It is worthy to mention that, in agreement with WHO recommendation, individuals that perform more than 150 minutes of physical activity per week are classified as physically active subjects with supposedly healthy benefits than those with less than 150 minutes of exercise per week [37]. Based on that, all participants of this study were properly considered physically active elderly individuals.

**Figure 3:**
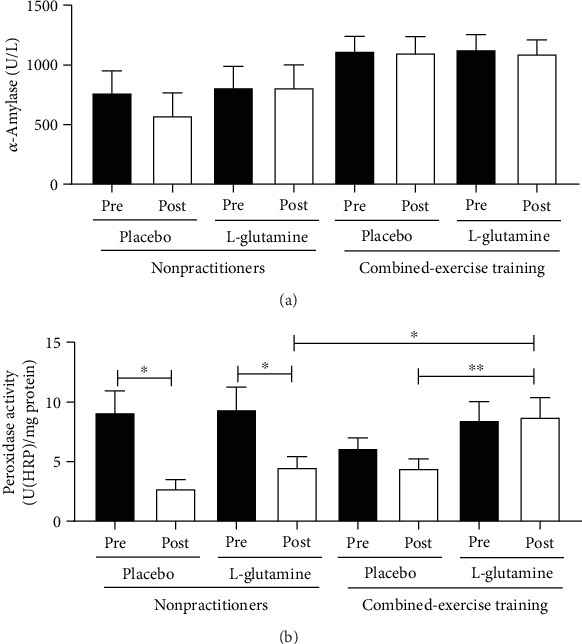
Results of enzymatic activity of *α*-amylase (U/L, a) and peroxidase (U(HRP)/mg protein, b) in the saliva of the elderly individuals in the NP and CET groups before (pre) and after 30 days (post) of supplementation with placebo or L-glutamine. Data were statistically analyzed using the two-way repeated measures ANOVA test and were presented as mean and standard deviation (SD) with a risk value of 5% (*p* < 0.05). ^∗^*p* < 0.05; ^∗∗^*p* < 0.01.

As shown in Figure 3, salivary *α*-amylase activity (Figure 3(a)) in the NP and CET groups was not statistically different before (pre) and after (post) supplementation with Gln or placebo. In a similar way, the salivary peroxidase activity (Figure 3(b)) found before (pre) supplementation period was not different between groups. However, after the supplementation period (post), NP volunteers supplemented with Gln or placebo showed significant reduction of the peroxidase activity compared to values found before (pre) supplementation (^∗^*p* < 0.05). In addition, higher peroxidase activity was observed in the saliva of CET group supplemented with Gln than the values found in the CET group supplemented with placebo (^∗∗^*p* < 0.01) or NP group supplemented with Gln (^∗^*p* < 0.05).

**Figure 4:**
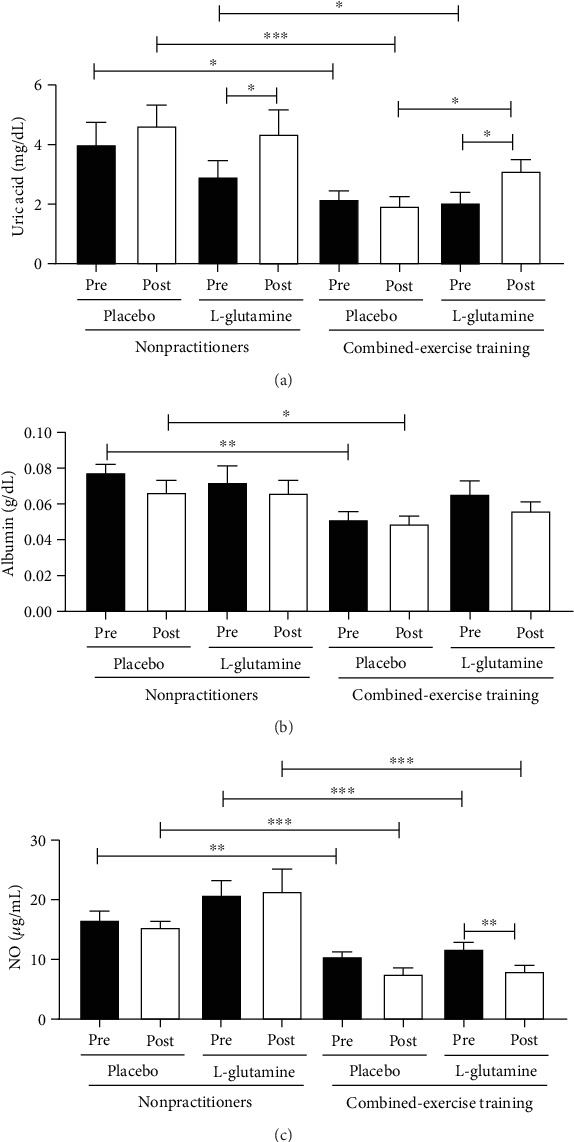
Salivary concentration of uric acid (mg/dL, a), albumin (g/dL, b), and nitric oxide (NO^·^*μ*g/mL, c) in the elderly individuals from the NP and CET groups before (pre) and 30 days after (post) daily supplementation with placebo or L-glutamine. Data were statistically analyzed using the two-way repeated measures ANOVA test and were presented as mean and standard deviation (SD) with a risk value of 5% (*p* < 0.05). ^∗^*p* < 0.05; ^∗∗^*p* < 0.01; ^∗∗∗^*p* < 0.001.

In Figure 4, it is showed that the salivary concentration of uric acid (Figure 4(a), ^∗^*p* < 0.05) and NO^·^ (Figure 4(c), ^∗∗^*p* < 0.01 and ^∗∗∗^*p* < 0.001) was higher before (pre) the supplementation period in the NP groups as compared to the values found in the CET groups, whereas the albumin concentration (Figure 4(b)) in the NP-PL group was higher only compared to the CET-PL group (^∗∗^*p* < 0.01). In relation to the data obtained 30 days after (post) daily supplementation with placebo or Gln, the salivary concentration of uric acid (Figure 4(a), ^∗∗^*p* < 0.001), albumin (Figure 4(b), ^∗^*p* < 0.05), and NO^·^ (Figure 4(c), ^∗∗∗^*p* < 0.001) in the NP groups was maintained higher than the CET groups. However, only the uric acid concentration found in the saliva from the NP-Gln group was not statistically different compared to the CET-Gln group. In addition, in the saliva of the volunteers of NP-Gln group, a higher uric acid concentration was observed when compared to the values found before the supplementation (^∗^*p* < 0.05), whereas the CET-Gln group showed increased salivary concentration of uric acid not only compared to the values found before supplementation period (^∗^*p* < 0.05) but also to the values observed in the CET-PL group (^∗^*p* < 0.05). Interestingly, reduction of salivary NO^·^ levels was observed in the CET-Gln group when compared to the values found before the supplementation period (^∗∗^*p* < 0.01).

As shown in Table 2, the values of Trolox-equivalent antioxidant capacity (TEAC), total protein content, and reducing power did not show significant differences between the volunteer groups (NP and CET) both before (pre) and after (post) supplementation with placebo or Gln. Regarding the salivary concentration of reduced glutathione (GSH), both volunteer groups supplemented with L-glutamine (NP-Gln and CET-Gln) showed significant lower concentrations postsupplementation, compared to the values found before (pre, *p* < 0.05). Oppositely, a significant reduction of the salivary concentration of oxidized glutathione (GSSG) was found after (post) supplementation period in both volunteer groups supplemented with placebo (NP-PL and CET-PL) when compared to the values observed before (pre, *p* < 0.05). In addition, the salivary GSSG concentration found that postsupplementation period in the NP-Gln group was lower than the values observed in the CET-Gln group (*p* < 0.05). Unaltered total protein content in saliva suggests similar secretory activity of the salivary glands between groups (*p* > 0.05).

Pearson's coefficient analysis (Table 3) showed a significant positive correlation between peroxidase activity and GSH concentration in the saliva of the volunteers from the CET-PL or CET-Gln groups before (pre) the supplementation period. Interestingly, whereas the CET-PL group did not maintain the correlation previously observed, the CET-Gln group not only sustained it but also increased those values found in the positive correlation between them. In addition, the same volunteers' group (CET-Gln) also presented a significant positive correlation between the peroxidase activity and uric acid concentration in the saliva after (post) the supplementation period.

**Figure 5:**
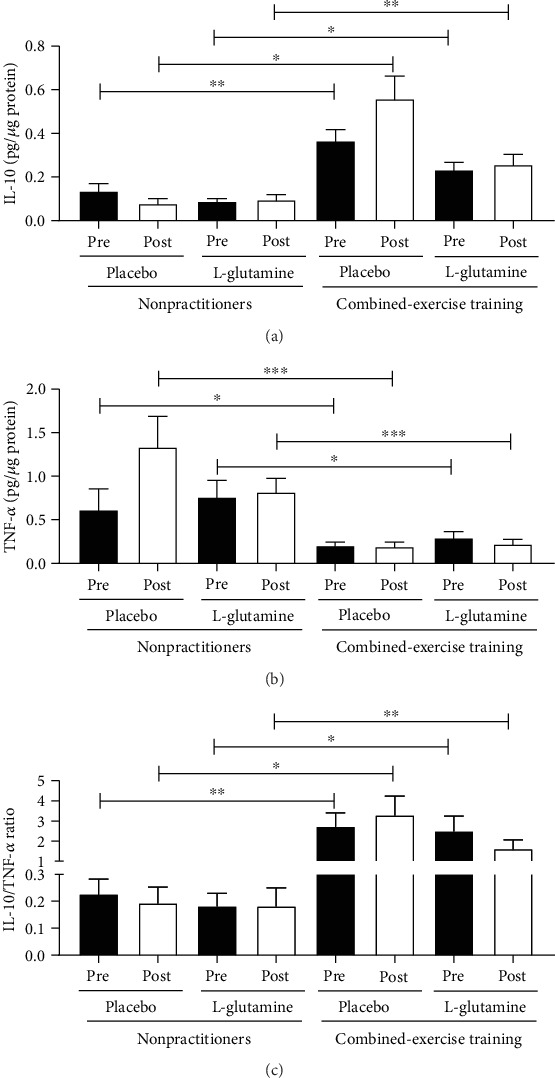
Salivary concentration of IL-10 (pg/*μ*g protein, a), TNF-*α* (pg/*μ*g protein, b), and IL-10/TNF-*α* ratio (c) in the elderly individuals from the NP and CET groups before (pre) and after 30 days (post) of daily supplementation with placebo or L-glutamine. Data were statistically analyzed using the two-way repeated measures ANOVA test and were presented as mean and standard deviation (SD) with a risk value of 5% (*p* < 0.05). ^∗^*p* < 0.05; ^∗∗^*p* < 0.01; ^∗∗∗^*p* < 0.001.

Figure 5 shows that the volunteers from the CET groups (supplemented with placebo or Gln) showed higher salivary IL-10 concentration both before (pre, ^∗^*p* < 0.05) and after (post, ^∗∗^*p* < 0.01) the supplementation period, compared with the values found in the NP groups at the same time points (Figure 5(a)). In contrast, the volunteers from the NP groups (supplemented with placebo or Gln) showed increased salivary TNF-*α* concentration both before (pre, ^∗^*p* < 0.05) and after (post, ^∗∗∗^*p* < 0.001) the supplementation period compared with the values found in the CET groups at the same time points (Figure 5(b)). In addition, the ratio between the salivary concentration of IL-10 and TNF-*α* (IL-10/TNF-*α*) showed higher values in the CET groups (supplemented with placebo or L-glutamine) than NP groups (Figure 5(c), ^∗^*p* < 0.05 and ^∗∗^*p* < 0.01) both before (pre) and after (post) the supplementation period.

## 4. Discussion

Our results showed that regular exercise activity, itself, induces significant decreases in background levels of uric acid and nitric oxide in both groups supplemented with placebo or L-glutamine, whereas background albumin levels in saliva were only altered by exercise in placebo aged subjects. In addition, lower background levels of NO^·^ and TNF-*α*, and also higher IL-10 levels, leading to increased IL-10/TNF-*α* ratio, were found in the saliva of exercising subjects compared to nonpractitioners. Regarding supplementation, our results showed higher background levels of uric acid in both groups supplemented with L-glutamine, whereas only exercising subjects plus L-glutamine showed lower NO^·^ levels. Besides, both groups supplemented with L-glutamine showed depleted GSH levels, while both placebo groups showed lower GSSG, after the supplementation period, compared to background values. Interestingly, lower peroxidase activity was found in both nonpractitioners after supplementation, compared to background levels. These results allow us to reinforce the ability of combined-exercise training to induce anti-inflammatory responses and improve redox homeostasis, and also, for the first time, that the L-glutamine supplementation is able to modulate these parameters in the oral environment.

It is widely accepted that bacterial pathologies, such as dental cavities and periodontitis could affect the redox condition in the oral compartment, even in the initial stages of infection [38]. However, no volunteer attested discomfort or noticed any alteration on their buccal health during the study, which minimizes the contribution from inflammatory/infectious processes on the observed salivary biomarkers. Moreover, we have also to consider that many other factors could also affect the redox balance in saliva, such as xenobiotics in atmospheric air (cigarette smoke, volatile aldehydes, polycyclic aromatic hydrocarbons), in food (acrolein, lipid hydroperoxides), or even some dental treatment procedures, such as ozone exposure, laser light, and ultraviolet light [39].

Saliva is provided with an arsenal of defense enzymes and compounds, most of them responsible for efficient immunological and antioxidant mechanisms in the oral cavity. Nagler et al. [40] have shown the prevalence of antioxidant capacity from the parotid saliva compared with the submandibular/sublingual saliva, based on peroxidase, superoxide dismutase, uric acid, and total antioxidant status. Due to experimental limitations, we cannot present the specific contribution from the submandibular or parotid glands in the total antioxidant capacity of saliva measured in our study. Instead, here, we were able to present an overall redox homeostasis and inflammatory response evaluation in saliva.

Aging has been associated with an overall oxidative stress condition in humans, which could be reasonably evidenced by accumulation of oxidative biomarkers (oxidized products) in several circulating cells, organs, and body fluids, such as saliva [41]. On the other hand, it has been well established that regular (moderate) exercises impose adaptive oxidative challenges to human body that improve the redox balance in several biological compartments, as determining factors for good health and quality of life for elderly population [42, 43]. Many health benefits are linked with redox hormesis and regular aerobic and/or resistance exercises, such as cardiovascular fitness [44], increased lean/fat mass ratio [45], lower risks of atherosclerosis and neuroinflammation [9], and improvement of cognitive functions [46]. Based on WHO, any physical activity is better than none at all [1]. In accordance, our results showed that the exercising subjects presented a better control of inflammatory response and redox balance, since lower background concentrations of two important antioxidants in the saliva—uric acid and albumin [47, 48]—associated with reduced NO^·^ levels could result from lower exposure to oxidative conditions in the buccal environment of exercising aged individuals. It is worthy to mention that oral NO^·^ production is mainly catalyzed by inducible nitric oxide synthase of activated neutrophils and resident macrophages (in the salivary glands) [49, 50]. Moreover, NO^·^ formation (by inducible nitric oxide synthase) is the rate-limiting step of peroxynitrite formation (ONOO^−^), a very reactive RNS in biological systems (k_1_~3 − 7  × 10^9^ M^−1^s^−1^) [49, 51]. In the buccal environment, ONOO^−^ decomposes, in a pH- and metal-dependent manner, to generate a wide range of secondary, but not less reactive, radicals, including hydroxyl (HO^·^), nitrogen dioxide (NO_2_^·^), and carbonate radical (CO_3_^·-^) [52, 53].

Therefore, the reduction of NO^·^ levels observed in exercising subjects can suggest that an anti-inflammatory condition was created in the oral environment, which was corroborated by higher ratios between the anti-inflammatory cytokine, IL-10, and the proinflammatory TNF-*α*. By controlling inflammation (based on IL-10/TNF-*α* ratios), further oxidative insults triggered by activated neutrophils and macrophages in the oral cavity were also prevented, which culminated in an overall maintenance of the redox balance in the saliva, as evidenced by unaltered antioxidant capacity (TEAC) and reducing power scores. In agreement, regular exercising aged individuals did not show different background *α*-amylase or peroxidase activities in saliva, compared with nonpractitioners.

Although regular exercise itself provides beneficial hormonal balance, promotes protein/enzyme turnover, and enhances immunological competence [54], the efficiency of antioxidant or ergogenic supplements remain controversial. Few solid meta-analysis studies were only able to sustain recommendation of high-protein or creatine supplementation in order to enhance muscle mass, strength, and functional performance in elderly, as a nutritional strategy to circumvent the harmful effects of sarcopenia [55–57]. Among several amino acid and antioxidants, L-glutamine supplementation emerged with prominent results of muscle mass/strength recovery, immune and inflammatory modulation, and redox balance in aging animal models and few aging human trials [15, 19, 58, 59].

Many studies involving L-glutamine supplementation have reported that exhaustive exercise depletes plasma L-glutamine concentrations, which could be directly associated to increased susceptibility to infections [60]. On the other hand, L-glutamine supplementation in regularly, moderate intensity, exercising individuals could result in long-term optimized protein synthesis in skeletal muscles [61] and glycogen resynthesize [62]. Furthermore, a strong correlation was already observed between L-glutamine supplementation and glutathione (GSH) levels in several body fluids, tissues, and cells in humans [63]. GSH is a *γ*-glutamyl-cysteinyl-glycine tripeptide strongly involved in the thiol-dependent antioxidant and redox-sensing network in living organism, both as a direct scavenger of ROS/RNS, or as substrate for several antioxidant enzymes, such as glutathione peroxidase and glutathione S-transferase [64].

Regarding the antioxidant balance in saliva of subjects here, 30-day L-glutamine supplementation significantly increased uric acid concentrations, in parallel to lower GSH levels, both in an exercise-independent manner. Focusing on the L-glutamine effect, itself, a remarkable increase of the peroxidase activity in saliva was only observed in L-glutamine-supplemented exercising subjects (CET-Gln), which sustains the hypothesis of an anti-inflammatory effect of L-glutamine/exercise combination in the saliva of aged individuals. Corroborating this fact, a positive correlation between the total peroxidase activity in the saliva and GSH levels in exercising aged individuals was observed before and after L-glutamine supplementation. Concerning the relationship between GSH levels and oxidant molecules in saliva, Maciejczyk et al. [49] showed a direct correlation between diminished GSH levels in both parotid and submandibular glands and oxidized lipid/protein adducts in the early stage of acute pancreatitis, although no information about peroxidase activity was provided in that study [49]. So, based on our results, we can suggest that, despite L-glutamine-related lower GSH concentrations in saliva, an improvement of the enzymatic/thiol-based antioxidant responses occurred in exercising aged individuals. Accordingly, uric acid and GSH were also positively correlated only in L-glutamine-exercised group of elderly individuals.

Interestingly, an unexpected decrease of peroxidase activity was observed in nonpractitioners pre/post placebo or L-glutamine supplementation. Unpredictable variables, such as minor variations of the peroxidase enzyme components lactoperoxidase, myeloperoxidase, or GSH/H_2_O_2_-peroxidase [65], eventual anxiety behavior from participants [66], or even changes in oral hygiene or unknown fluoride treatment [36] could inadvertently cause the observed decreases in salivary peroxidase activity of participants herewith.

Several studies have shown that dietary L-glutamine supplementation reduces plasma nitrite/nitrate levels, indicating that L-glutamine has an impact on L-arginine-NO^·^ metabolism (by inhibiting inducible nitric oxide synthase) [67, 68]. Accordingly, the L-glutamine supplementation of aging exercising subjects caused a significant decrease of NO^·^ levels in their saliva, suggesting a prominent pre/post anti-inflammatory effect, even unchanging the levels of IL-10 and TNF-*α* in the saliva of the L-glutamine-exercise group.

Nevertheless, regular (moderate) exercises, following the well-accepted redox hormesis principle, has been argued as a nonpharmacological intervention to promote health in many human subgroups, including the elderly population, as shown here [69].

## 5. Conclusion

Taken together, our results in saliva showed that the combined-exercise training was able to modulate both inflammatory responses and redox balance in elderly individuals and also, for the first time, that the daily supplementation with L-glutamine for 30 days improved the benefits provided by the exercise program on these remarkable parameters associated to healthy aging.

## Figures and Tables

**Table 1:** Physical (means ± standard deviation (SD)) characteristics of elderly individuals from the NP and CET groups before (pre) and after 30 days (post) of daily supplementation. Significance level of ^∗^*p* < 0.05.

Characteristics	Volunteers (*n* = 54)				*p* value
	Nonpractitioners (NP, *n* = 34)		Combined-exercise training (CET, *n* = 49)		
	Placebo (*n* = 15)	L-glutamine (*n* = 19)	Placebo (*n* = 15)	L-glutamine (*n* = 19)	
Age (year)	73.5 ± 7.7	73.1 ± 5.9	72.1 ± 6.0	71.6 ± 5.8	>0.05
Height (m)	1.55 ± 0.1	1.59 ± 0.09	1.56 ± 0.10	1.57 ± 0.09	>0.05
Weight (kg)	65.1 ± 11.1	73.2 ± 14.5^∗^	62.5 ± 10.2	63.2 ± 12.9	<0.05
Body mass index (kg/m^2^)	27.1 ± 3.1	28.7 ± 4.2^∗^	25.5 ± 3.5	25.5 ± 4.0	<0.05
Total body fat (%)	37.6 ± 8.7	40.5 ± 9.0	35.5 ± 5.4	36.2 ± 8.4	>0.05
Fat-free mass (%)	58.7 ± 7.6	59.9 ± 8.7	62.8 ± 7.3	64.6 ± 6.7	>0.05
Skeletal muscle mass (kg)	20.1 ± 5.1	19.8 ± 4.5	19.2 ± 2.7	19.7 ± 3.7	>0.05
IPAQ					
Physical activity (min/week)	908 ± 212^#^	956 ± 208^#^	1894 ± 252	1961 ± 253	<0.05
Sitting (min/week)	1705 ± 440^#^	1977 ± 587^#^	1312 ± 463	1414 ± 535	<0.05

^∗^Significant differences in comparison to values obtained in the other groups. ^#^Significant differences in comparison to values obtained in the CET groups.

**Table 2:** Salivary concentration of TEAC (Trolox-equivalent antioxidant capacity, *μ*mol eq. Trolox/mg protein), reduced glutathione (GSH, *μ*M), oxidized glutathione (GSSG, *μ*M), reducing power (A.U.), and total protein (mg/mL) in the elderly individuals from the NP and CET groups before (Pre) and after 30 days (Post) of daily supplementation with placebo or L-glutamine. Data were statistically analyzed using the Two-way repeated measures ANOVA test and were presented as mean and standard deviation (SD) with a risk value of 5% (*p* < 0.05).

Groups	Nonpractitioners (NP, *n* = 34)				Combined-exercise training (CET, *n* = 49)			
	Placebo (*n* = 15)		L-glutamine (*n* = 19)		Placebo (*n* = 26)		L-glutamine (*n* = 23)	
Variables	Pre	Post	Pre	Post	Pre	Post	Pre	Post
TEAC (*μ*mol eq. trolox/mg proteins)	0.45 ± 0.09	0.42 ± 0.14	0.54 ± 0.09	0.51 ± 0.14	0.46 ± 0.08	0.51 ± 0.09	0.40 ± 0.05	0.35 ± 0.05
GSH (*μ*M)	400.5 ± 83.67	389.1 ± 88.31	488.8 ± 66.84^∗^	309.1 ± 36.57	381.6 ± 43.46	345.0 ± 45.23	384.3 ± 59.38∗	242.6 ± 38.96
GSSG (*μ*M)	65.8 ± 12.03^∗^	42.43 ± 9.42	49.26 ± 9.01	33.65 ± 5.56^#^	86.04 ± 11.8^∗^	53.94 ± 7.78	57.18 ± 11.2	66.69 ± 11.1
Reducing power (A.U.)	0.71 ± 0.06	0.70 ± 0.07	0.74 ± 0.05	0.72 ± 0.05	0.68 ± 0.04	0.66 ± 0.04	0.77 ± 0.03	0.69 ± 0.04
Total protein content (mg/mL)	1.64 ± 0.42	1.61 ± 0.41	1.62 ± 0.37	1.49 ± 0.47	1.71 ± 0.33	1.59 ± 0.33	1.54 ± 0.38	1.72 ± 0.34

^∗^Significant differences in comparison to values obtained in the pre and postsupplementation period (*p* < 0.05). ^#^Significant differences in comparison to values obtained in the nonpractitioners and combined-exercise training groups (*p* < 0.05).

**Table 3:** Results of correlation between peroxidase activity and reduced glutathione (GSH), oxidized glutathione (GSSG), reducing power (A.U.), and uric acid and correlation between uric acid and GSH, GSSG, and reducing power of elderly individuals from the NP and CET groups before (pre) and after 30 days (post) of daily supplementation with placebo or L-glutamine. Data were statistically analyzed using Pearson's correlation with a risk value of 5% (*p* < 0.05).

Groups	Nonpractitioners (*n* = 34)				Combined-exercise training (*n* = 51)			
	Placebo (*n* = 15)		L-glutamine (*n* = 19)		Placebo (*n* = 26)		L-glutamine (*n* = 25)	
Variables	Pre	Post	Pre	Post	Pre	Post	Pre	Post
Peroxidase activity by GSH	*r* = 0.14, *p* = 0.64	*r* = −0.37, *p* = 0.25	*r* = −0.07, *p* = 0.79	*r* = −0.12, *p* = 0.65	*r* = 0.40, *p* = 0.04	*r* = 0.04, *p* = 0.84	*r* = 0.63, *p* = 0.002	*r* = 0.69, *p* = 0.001
Peroxidase activity by GSSG	*r* = −0.41, *p* = 0.12	*r* = −0.45, *p* = 0.19	*r* = 0.09, *p* = 0.73	*r* = 0.36, *p* = 0.18	*r* = −0.06, *p* = 0.74	*r* = 0.08, *p* = 0.71	*r* = 0.25, *p* = 0.29	*r* = −0.05, *p* = 0.81
Peroxidase activity by reducing power	*r* = −0.05, *p* = 0.84	*r* = −0.20, *p* = 0.54	*r* = −0.27, *p* = 0.32	*r* = −0.41, *p* = 0.12	*r* = 0.34, *p* = 0.08	*r* = 0.01, *p* = 0.95	*r* = −0.40, *p* = 0.09	*r* = 0.25, *p* = 0.26
Peroxidase activity by uric acid	*r* = 0.46, *p* = 0.09	*r* = −0.44, *p* = 0.19	*r* = 0.01, *p* = 0.96	*r* = −0.11, *p* = 0.70	*r* = 0.15, *p* = 0.53	*r* = 0.23, *p* = 0.34	*r* = 0.31, *p* = 0.13	*r* = 0.75, *p* = 0.0001
Uric acid by GSH	*r* = −0.01, *p* = 0.97	*r* = 0.42, *p* = 0.12	*r* = −0.33, *p* = 0.16	*r* = −0.34, *p* = 0.15	*r* = −0.05, *p* = 0.81	*r* = −0.25, *p* = 0.24	*r* = −0.12, *p* = 0.60	*r* = 0.07, *p* = 0.77
Uric acid by GSSG	*r* = −0.17, *p* = 0.55	*r* = −0.28, *p* = 0.34	*r* = −0.18, *p* = 0.48	*r* = −0.43, *p* = 0.06	*r* = −0.34, *p* = 0.09	*r* = −0.23, *p* = 0.29	*r* = −0.22, *p* = 0.37	*r* = 0.11, *p* = 0.62
Uric acid by reducing power	*r* = −0.19, *p* = 0.49	*r* = 0.42, *p* = 0.12	*r* = −0.07, *p* = 0.80	*r* = 0.25, *p* = 0.31	*r* = 0.1, *p* = 0.63	*r* = 0.03, *p* = 0.90	*r* = −0.11, *p* = 0.63	*r* = 0.44, *p* = 0.04

## Data Availability

The data used to support the findings of this study are included within the article.
